# Lung and lupus vulgaris

**DOI:** 10.4103/0970-2113.80327

**Published:** 2011

**Authors:** V. Mukta, K. Jayachandran

**Affiliations:** *Department of Medicine, PSGIMS and R, Peelamedu, Coimbatore, Tamil Nadu, India*

**Keywords:** Lupus vulgaris, pleural effusion, tuberculosis

## Abstract

Lupus vulgaris is chronic, postprimary, paucibacillary cutaneous tuberculosis found in individuals with moderate immunity and high degree of tuberculin sensitivity. Eighty percent of the lesions are on the head and neck. We present the case of a 38 year old lady who was admitted with complaints of worsening breathlessness and low grade fever of one month duration. Examination showed multiple, nontender skin ulcers on bilateral lumbar areas, two oozing serosanguinous discharge and others scarred in the centre. Respiratory system examination and chest X-ray revealed right sided pleural effusion. On investigation, pleural fluid was tuberculous in nature. Skin biopsy from the edge of ulcer was also suggestive of tuberculosis. Patient is doing well on antituberculous drugs. This case highlights the importance of cutaneous manifestations of systemic disease and is an example of the unusual presentation of lupus vulgaris in a case of pleural effusion.

## CASE REPORT

**Figure 1:**
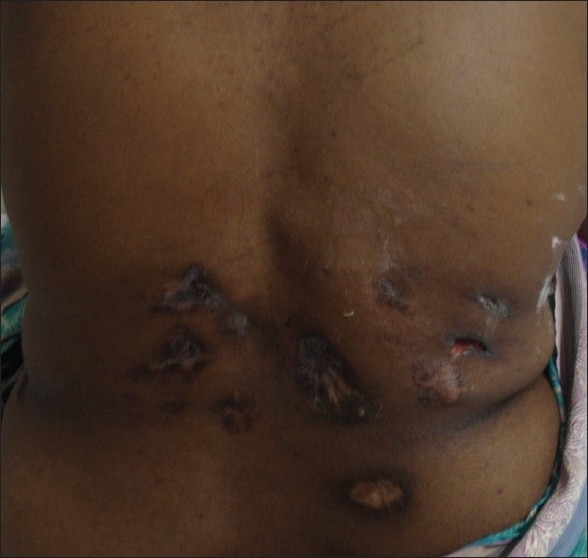
Cutaneous lesions on the back of patient

A thirty eight year old lady was admitted with complaints of worsening breathlessness and low grade fever of one month duration. This was associated with pleuritic type of chest pain on inspiration and orthopnea for past three days. She had developed multiple skin ulcers on her lower back over one month duration. Two of these were discharging serous fluid over past two weeks. She had undergone right knee surgery seven years ago and incisional hernia repair three years ago. There was no history suggestive of tuberculosis in the past. She had been treated unsuccessfully with antibiotics for suspected folliculitis with abscesses on the back, at another centre for one week prior to admission in our hospital. On examination, BCG vaccine scar was seen on the left deltoid. There were multiple, nontender skin ulcers on bilateral lumbar areas, some oozing serosanguinous discharge and few others with scarring [Figure 1]. Respiratory system examination revealed restricted right sided respiratory movements, stony dull percussion note in right hemithorax and absent breath sounds on right side in all lung fields. Other system examinations were normal.

**Figure 2:**
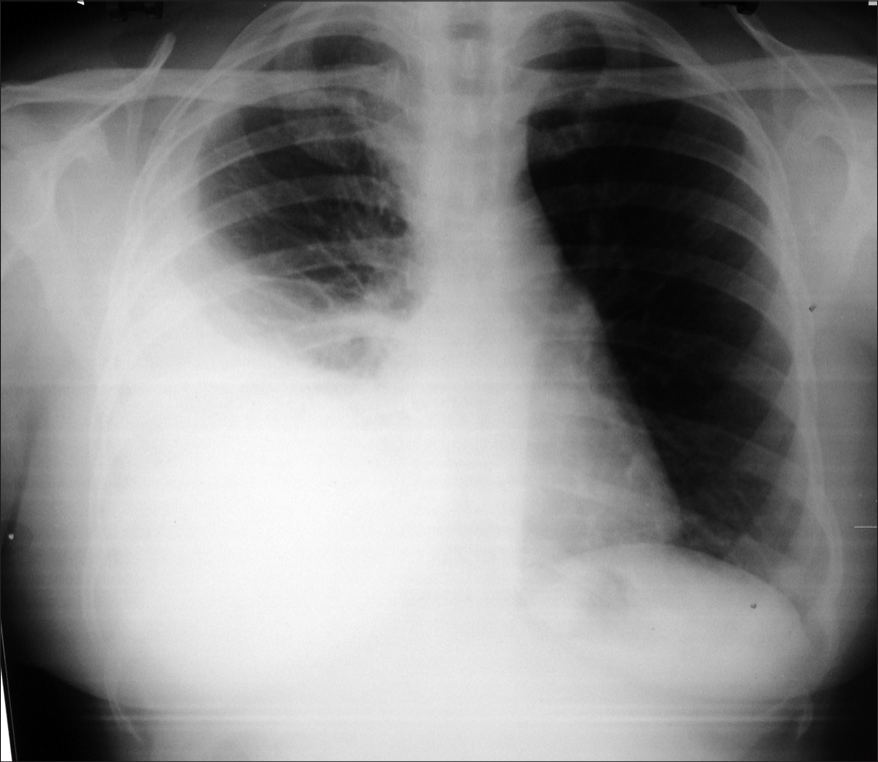
Chest X-ray of patient showing pleural effusion

**Figure 3:**
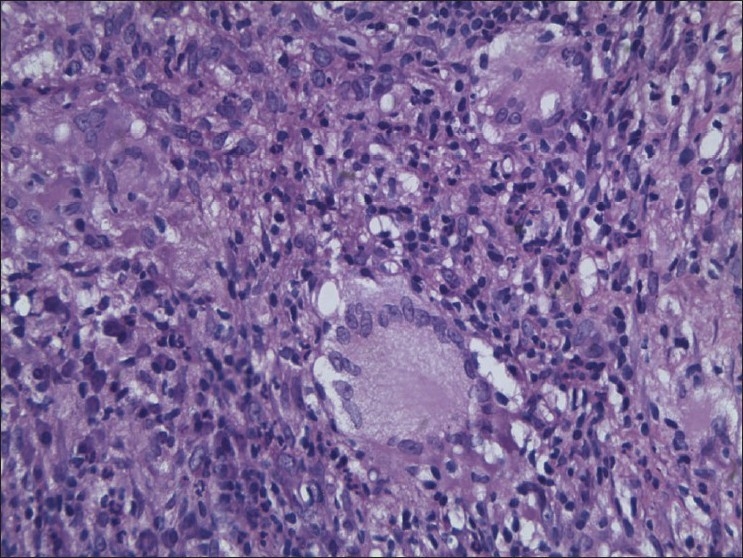
Histopathological slide photomicrograph (40×) showing granulomas

Chest X-ray was suggestive of right sided pleural effusion [Figure 2]. Pleural fluid aspiration and analysis was done. Results are detailed in Table 1. Pleural fluid was exudative in nature with 99% lymphocytes and adenosine deaminase level of 108 IU/L, suggestive of tuberculous etiology. Other blood investigation results are given in Table 2. Echocardiography was normal. Intradermal Mantoux (1 TU PPD with RT 23 Tween 80) was positive (12 mm) at the end of 48 hours. Skin biopsy was taken from the edge of the ulcer and histopathological examination was done. It was reported as granulomatous inflammation suggestive of tuberculosis. The biopsy specimen contained multiple granulomas composed of epithelial cells and langhan’s giant cells; areas of necrosis and dense lymphocytic infiltrate [Figure 3]. It did not stain for acid fast bacilli. Diagnosis of pulmonary tuberculosis with lupus vulgaris was made. Patient was started on antituberculous drugs. Pleural effusion and the skin lesions have regressed after eight weeks of antituberculous therapy. Presently she is on rifampicin and isoniazid, continuation phase, for the next four months.

**Table 1:** Pleural fluid analysis results

Pleural fluid	Straw coloured
Total count	733 cells /cu.mm
Cell count	99% were lymphocytes
Pleural fluid protein	7.4 g/dl,
Pleural fluid albumin	2.8g/dl
Pleural fluid glucose	157mg/dl
Pleural fluid lactate dehydrogenase	252 U/L
Pleural fluid adenosine deaminase	108IU/L (normal- <40)
Pleural fluid culture	Sterile
Pleural fluid malignant cytology	Negative

**Table 2:** Results of blood investigations

HIV ELISA	Negative
TSH	1.41 microU/ml
FT4	1.38ng/dl (0.932-1.71)
T3	0.968ng/ml (0.846-2.02)
Serum total protein	9.2 g/dl (6-10)
Serum albumin	3.4g/dl (3.4-4.8)
Globulin	5.8g/dl (1.8-3.6)
Alkaline phosphatase	182U/L (35-304)
AST	29U/L (upto 37)
ALT	31U/L (upto 40)
Bilirubin	0.2mg/dl (0.3-1.0)
Direct	0.1mg/dl (0.1-0.3)
LDH	195U/L (135-214)
GGT	88U/L (10-66)

## DISCUSSION

Incidence of cutaneous tuberculosis is 1-3/1000 persons in India.[1] Cutaneous tuberculosis is classified into primary and secondary forms.[2] Primary infection in a previously uninfected host can present as a chancre (direct inoculation) or acute disseminated miliary tuberculosis (hematogenous spread). Secondary cutaneous tuberculosis follows either reinfection or reactivation.[2] Lupus vulgaris and tuberculosis verrrucosa cutis are forms of reinfection tuberculosis and often occur in presensitised patients, by exogenous inoculation. Reactivation tuberculosis includes scrofuloderma and tuberculosis cutis orificialis.[2] Scrofuloderma occurs due to contiguous spread from underlying lymph node, bone, joint or epididymis, in patients with lowered cell mediated immunity. Tuberculosis cutis orificialis involve orifices like nose, mouth and anus, draining an active tuberculous infection.[2]

Lupus vulgaris is the most common form of cutaneous tuberculosis. Eighty percent of the lesions are on the head and neck.[3] Females are affected two to three times more often than males. It is caused by mycobacterium tuberculosis and can involve the skin by hematogenous or lymphatic route. Lupus vulgaris skin lesions are of five types - a) plaque b) ulcerative or mutilating c) vegetating d) tumour-like e) papulonodular. Atrophic scarring of lesions and apple jelly colour on diascopy are characteristic. Histopathologically, it is associated with non necrotizing granulomas in which acid fast bacilli are usually not found.[4] Summarizing, lupus vulgaris is chronic, postprimary, paucibacillary cutaneous tuberculosis found in individuals with moderate immunity and high degree of tuberculin sensitivity.[4] The disease treatment consists of systemic antituberculous drugs.

Our patient had pleural effusion with lupus vulgaris on the lower back (atypical site). She had presented with dyspnea and lupus vulgaris was suspected after general examination. This case highlights the need of awareness regarding cutaneous tuberculosis among physicians who manage pulmonary and extrapulmonary tuberculosis, especially in the era of HIV-associated opportunistic infections.
